# 4-Nitro-*N*-(3-nitro­phen­yl)benzamide

**DOI:** 10.1107/S1600536811038062

**Published:** 2011-09-30

**Authors:** Dean H. Johnston, Colin R. Taylor

**Affiliations:** a Department of Chemistry, Otterbein University, Westerville, OH 43081, USA

## Abstract

The title compound, C_13_H_9_N_3_O_5_, prepared as a solid derivative of 3-nitro­analine *via* reaction with 4-nitro­benzoyl chloride, crystallizes in a chiral space group. The mol­ecule is non-planar with a dihedral angle of 26.1 (1)° between the two benzene rings. Both nitro groups are twisted slightly out of the plane of their corresponding benzene rings, making dihedral angles of 10.7 (4) and 13.5 (4)°. The mol­ecules are stacked along the *a* axis with benzene ring centroid–centroid distances of 3.8878 (6) Å. In the crystal, inter­molecular benzene C—H⋯O inter­actions involving one nitro group and the carbonyl group link the mol­ecules, forming chains along [001]. An additional set of aromatic C—H⋯O inter­actions with the second nitro group form chains along [101], connecting adjacent chains to create layers perpendicular to the *b* axis.

## Related literature

For the preparation, properties and applications of the title compound, see: Kichitaro (1954 ▶); Shchel’tsyn *et al.* (1972 ▶); Kang *et al.* (2008 ▶). For related structures, see: Hariharan & Srinivasan (1990 ▶); Adams *et al.* (2001 ▶); Novozhilova *et al.* (1986 ▶); Sun *et al.* (2009 ▶). The title compound represents a relatively unusual example of an achiral mol­ecule in a chiral (Sohncke) space group with the conformational flexibility to convert to its mirror image (Pidcock, 2005 ▶).
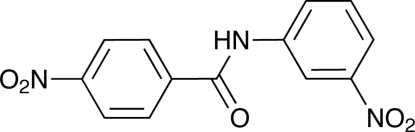


## Experimental

### 

#### Crystal data

C_13_H_9_N_3_O_5_
*M* *_r_* = 287.23
Monoclinic, 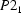

*a* = 3.8878 (6) Å
*b* = 21.686 (3) Å
*c* = 7.3919 (11) Å
β = 90.294 (11)°
*V* = 623.20 (16) Å^3^
*Z* = 2
Mo *K*α radiation
μ = 0.12 mm^−1^
*T* = 200 K
0.45 × 0.20 × 0.12 mm

#### Data collection

Bruker SMART X2S benchtop diffractometer
Absorption correction: multi-scan (*SADABS*; Bruker, 2009 ▶) *T*_min_ = 0.737, *T*_max_ = 0.989
3940 measured reflections
1136 independent reflections
1008 reflections with *I* > 2σ(*I*)
*R*_int_ = 0.032

#### Refinement

*R*[*F* ^2^ > 2σ(*F* ^2^)] = 0.034
*wR*(*F* ^2^) = 0.083
*S* = 1.07
1136 reflections
190 parameters
1 restraint
H-atom parameters constrained
Δρ_max_ = 0.13 e Å^−3^
Δρ_min_ = −0.17 e Å^−3^

### 

Data collection: *APEX2* and GIS (Bruker, 2009 ▶); cell refinement: *SAINT* (Bruker, 2009 ▶); data reduction: *SAINT*; program(s) used to solve structure: *SHELXS97* (Sheldrick, 2008 ▶); program(s) used to refine structure: *SHELXL97* (Sheldrick, 2008 ▶) and *OLEX2* (Dolomanov *et al.*, 2009 ▶); molecular graphics: *PLATON* (Spek, 2009 ▶), *Mercury* (Macrae *et al.*, 2008 ▶), and *POV-RAY* (Cason, 2004 ▶); software used to prepare material for publication: *publCIF* (Westrip, 2010 ▶).

## Supplementary Material

Crystal structure: contains datablock(s) I, global. DOI: 10.1107/S1600536811038062/zl2389sup1.cif

Structure factors: contains datablock(s) I. DOI: 10.1107/S1600536811038062/zl2389Isup2.hkl

Supplementary material file. DOI: 10.1107/S1600536811038062/zl2389Isup3.mol

Supplementary material file. DOI: 10.1107/S1600536811038062/zl2389Isup4.cml

Additional supplementary materials:  crystallographic information; 3D view; checkCIF report

## Figures and Tables

**Table 1:** Hydrogen-bond geometry (Å, °)

*D*—H⋯*A*	*D*—H	H⋯*A*	*D*⋯*A*	*D*—H⋯*A*
C2—H2⋯O1^i^	0.95	2.50	3.375 (4)	153
C5—H5⋯O3^ii^	0.95	2.38	3.263 (4)	155
C13—H13⋯O5^iii^	0.95	2.51	3.421 (4)	162

Symmetry codes: (i) 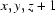; (ii) 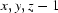; (iii) 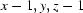.

**Fig. 1.**
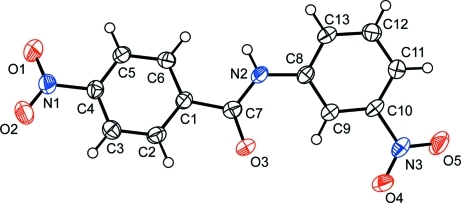
The molecular structure of the title compound with the atom labeling scheme drawn with 50% probability displacement ellipsoids for non-H atoms.

**Fig. 2.**
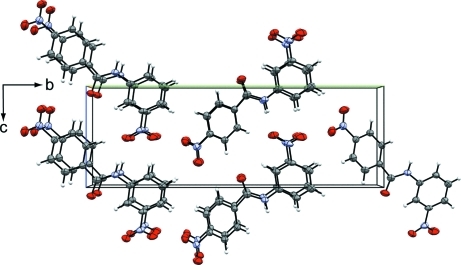
The packing of the title compound viewed down the a axis drawn with 50% probability displacement ellipsoids for non-H atoms.

**Fig. 3.**
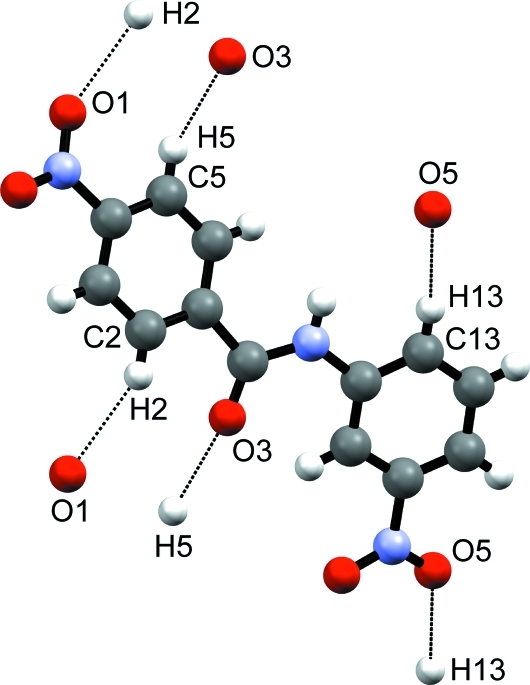
Ball and stick diagram of the hydrogen bonding interactions of the title compound with participating atoms labeled. For operators for symmetry created atoms (omitted here) see Table 1.

**Fig. 4.**
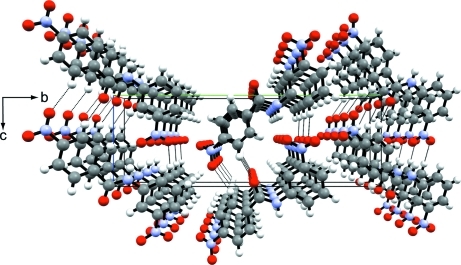
Perspective view down the a axis of the packing of molecules with hydrogen bonding interactions represented by dashed lines.

**Fig. 5.**
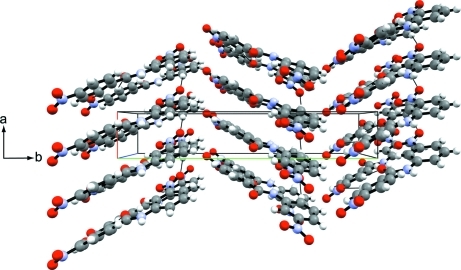
Perspective view down the c axis of the packing of molecules with hydrogen bonding interactions represented by dashed lines. Hydrogen bonds connect molecules along [001] (the viewing axis) and [101].

## supplementary crystallographic information

### Comment

The title compound was prepared as a solid derivative of 3-nitroanaline for a qualitative organic analysis laboratory course. The starting material was 3-nitroaniline, and reaction with 4-nitrobenzoyl chloride produced a *p*-nitrobenzamide derivative.

The title compound crystallizes in the chiral spacegroup *P*2_1_ and represents a relatively unusual example of an achiral molecule in a chiral (Sohncke) space group with the conformational flexibility to convert to its mirror image (Pidcock, 2005).

The molecule (Fig. 1) is non-planar with a dihedral angle of approximately 26.1 (1)° between the two aromatic rings. Both nitro groups are twisted slightly out of the plane of their corresponding aromatic rings, with dihedral angles of 10.7 (4) (N1, O1, O2) and 13.5 (4) (N3, O4, O5) degrees.

In the unit cell, the molecules are stacked along the *a* axis (Fig. 2) with aromatic ring centroid-centroid distances of 3.8878 (6) Å, corresponding precisely to the length of the *a* axis. The ring numbered C1–C6 stacks with a plane-centroid distance of 3.392 (2) Å and a ring shift of 1.899 (4) Å. The ring numbered C8–C13 stacks with a plane-centroid distance of 3.483 (2) Å and a ring shift of 1.728 (4) Å.

The title compound forms hydrogen bonding interactions with adjacent molecules along two different axes to create layers perpendicular to the *b* axis. The C2—H2···O1^i^ and C5—H5···O3^ii^ interactions (Fig. 3, Fig. 4, Table 1) form chains along [001] (all symmetry operators as in Table 1). Additional C13—H13···O5^iii^ interactions also connect adjacent molecules, with the resulting chains running along [101] (Fig. 5). The position of the N—H in the molecule prevents it from forming a significant hydrogen bonding interaction (H2N···O4^iii^ distance of 2.65 Å, greater than the sum of the van der Waals radii).

### Experimental

Approximately 1.0 g (7.24 mmol) of 3-nitroaniline was dissolved in 3.0 ml of pyridine in a small test tube. To this was added 0.5 g (2.70 mmol) of 4-nitrobenzoyl chloride. This mixture was warmed slightly with a water bath until homogeneous, allowed to cool to room temperature, and then poured into 10.0 ml of water. The solution was allowed to separate and the top layer was decanted. The residue was stirred with 5.0 ml of a 5% Na_2_CO_3_ solution, and then cooled in an ice bath to induce crystallization. The crude crystals were filtered, and then recrystallized from absolute ethanol to produce 4-nitro-*N*-(3-nitrophenyl)benzamide, mp = 502–503 K (lit = 500–501 K, Kang *et al.* (2008).)

### Refinement

All hydrogen atoms were located in difference maps and refined with the atom positions constrained to the external bisector of the appropriate *X*—C—Y or *X*—N—Y atom with C—H distances of 0.95 Å and an N—H distance of 0.88 Å. A riding model was used for all H atoms with *U*_iso_(H) = 1.2 times *U*_iso_(C) or *U*_iso_(N). In the absence of significant anomalous scattering effects Friedel pairs were merged in the final refinement.

### Crystal data

**Table:**

C_13_H_9_N_3_O_5_	*D*_x_ = 1.531 Mg m^−^^3^
*M**_r_* = 287.23	Melting point: 502 K
Monoclinic, *P*2_1_	Mo *K*α radiation, λ = 0.71073 Å
*a* = 3.8878 (6) Å	Cell parameters from 1373 reflections
*b* = 21.686 (3) Å	θ = 2.8–24.6°
*c* = 7.3919 (11) Å	µ = 0.12 mm^−^^1^
β = 90.294 (11)°	*T* = 200 K
*V* = 623.20 (16) Å^3^	Block, clear colourless
*Z* = 2	0.45 × 0.20 × 0.12 mm
*F*(000) = 296	

### Data collection

**Table:**

Bruker SMART X2S benchtop diffractometer	1136 independent reflections
Radiation source: fine-focus sealed tube	1008 reflections with *I* > 2σ(*I*)
doubly curved silicon crystal	*R*_int_ = 0.032
Detector resolution: 8.3330 pixels mm^-1^	θ_max_ = 25.1°, θ_min_ = 2.8°
ω scans	*h* = −4→3
Absorption correction: multi-scan (*SADABS*; Bruker, 2009)	*k* = −25→25
*T*_min_ = 0.737, *T*_max_ = 0.989	*l* = −8→8
3940 measured reflections	

### Refinement

**Table:**

Refinement on *F*^2^	Primary atom site location: structure-invariant direct methods
Least-squares matrix: full	Secondary atom site location: difference Fourier map
*R*[*F*^2^ > 2σ(*F*^2^)] = 0.034	Hydrogen site location: difference Fourier map
*wR*(*F*^2^) = 0.083	H-atom parameters constrained
*S* = 1.07	*w* = 1/[σ^2^(*F*_o_^2^) + (0.046*P*)^2^ + 0.048*P*] where *P* = (*F*_o_^2^ + 2*F*_c_^2^)/3
1136 reflections	(Δ/σ)_max_ < 0.001
190 parameters	Δρ_max_ = 0.13 e Å^−^^3^
1 restraint	Δρ_min_ = −0.17 e Å^−^^3^

### Special details

**Table:**

Geometry. All e.s.d.'s (except the e.s.d. in the dihedral angle between two l.s. planes) are estimated using the full covariance matrix. The cell e.s.d.'s are taken into account individually in the estimation of e.s.d.'s in distances, angles and torsion angles; correlations between e.s.d.'s in cell parameters are only used when they are defined by crystal symmetry. An approximate (isotropic) treatment of cell e.s.d.'s is used for estimating e.s.d.'s involving l.s. planes.
Refinement. Refinement of *F*^2^ against ALL reflections. The weighted *R*-factor *wR* and goodness of fit *S* are based on *F*^2^, conventional *R*-factors *R* are based on *F*, with *F* set to zero for negative *F*^2^. The threshold expression of *F*^2^ > σ(*F*^2^) is used only for calculating *R*-factors(gt) *etc*. and is not relevant to the choice of reflections for refinement. *R*-factors based on *F*^2^ are statistically about twice as large as those based on *F*, and *R*- factors based on ALL data will be even larger.

### Fractional atomic coordinates and isotropic or equivalent isotropic displacement parameters (Å^2^)

**Table:**

	*x*	*y*	*z*	*U*_iso_*/*U*_eq_	
C1	0.5757 (8)	0.50143 (14)	0.7761 (4)	0.0293 (7)	
C2	0.4065 (8)	0.55485 (16)	0.8360 (4)	0.0342 (8)	
H2	0.3754	0.5616	0.9618	0.041*	
C3	0.2833 (8)	0.59829 (15)	0.7109 (4)	0.0335 (7)	
H3	0.1647	0.6342	0.7499	0.040*	
C4	0.3402 (8)	0.58731 (15)	0.5270 (4)	0.0305 (7)	
C5	0.5099 (8)	0.53518 (14)	0.4631 (4)	0.0324 (7)	
H5	0.5439	0.5292	0.3372	0.039*	
C6	0.6291 (8)	0.49190 (14)	0.5889 (4)	0.0310 (7)	
H6	0.7462	0.4560	0.5487	0.037*	
N1	0.2079 (8)	0.63348 (12)	0.3938 (4)	0.0376 (7)	
O1	0.3012 (8)	0.62833 (13)	0.2344 (3)	0.0634 (9)	
O2	0.0141 (7)	0.67441 (11)	0.4478 (3)	0.0505 (7)	
C7	0.7131 (8)	0.45847 (15)	0.9221 (4)	0.0331 (7)	
O3	0.7597 (8)	0.47773 (12)	1.0776 (3)	0.0533 (7)	
N2	0.7873 (7)	0.39887 (12)	0.8706 (3)	0.0333 (6)	
H2N	0.7336	0.3889	0.7585	0.040*	
C8	0.9407 (8)	0.35147 (14)	0.9776 (4)	0.0288 (7)	
C9	1.0551 (8)	0.36141 (15)	1.1561 (4)	0.0300 (7)	
H9	1.0295	0.4004	1.2133	0.036*	
C10	1.2070 (8)	0.31193 (15)	1.2457 (4)	0.0293 (7)	
C11	1.2552 (8)	0.25372 (14)	1.1709 (4)	0.0317 (7)	
H11	1.3622	0.2214	1.2371	0.038*	
C12	1.1382 (9)	0.24514 (16)	0.9931 (4)	0.0352 (8)	
H12	1.1654	0.2061	0.9368	0.042*	
C13	0.9830 (8)	0.29302 (14)	0.8981 (4)	0.0318 (7)	
H13	0.9045	0.2862	0.7778	0.038*	
N3	1.3347 (7)	0.32240 (13)	1.4340 (3)	0.0344 (6)	
O4	1.2424 (7)	0.36973 (11)	1.5145 (3)	0.0508 (7)	
O5	1.5281 (7)	0.28377 (13)	1.5016 (3)	0.0543 (8)	

### Atomic displacement parameters (Å^2^)

**Table:**

	*U*^11^	*U*^22^	*U*^33^	*U*^12^	*U*^13^	*U*^23^
C1	0.0323 (17)	0.0299 (17)	0.0255 (14)	−0.0032 (15)	−0.0023 (13)	0.0021 (12)
C2	0.0383 (18)	0.0392 (19)	0.0253 (14)	0.0005 (16)	0.0027 (13)	−0.0022 (13)
C3	0.0303 (17)	0.0330 (18)	0.0371 (17)	0.0016 (15)	0.0012 (13)	−0.0011 (14)
C4	0.0320 (16)	0.0287 (17)	0.0308 (16)	−0.0020 (14)	−0.0026 (12)	0.0035 (13)
C5	0.0403 (18)	0.0312 (18)	0.0257 (15)	−0.0023 (15)	−0.0015 (13)	−0.0001 (13)
C6	0.0346 (18)	0.0283 (17)	0.0301 (15)	0.0007 (14)	0.0000 (13)	−0.0031 (12)
N1	0.0463 (18)	0.0298 (15)	0.0367 (15)	0.0001 (14)	−0.0049 (13)	0.0033 (13)
O1	0.099 (2)	0.0583 (19)	0.0333 (14)	0.0236 (17)	0.0009 (14)	0.0090 (13)
O2	0.0610 (17)	0.0369 (15)	0.0537 (15)	0.0135 (14)	0.0044 (13)	0.0066 (12)
C7	0.0341 (18)	0.037 (2)	0.0277 (16)	0.0001 (15)	0.0005 (13)	−0.0016 (13)
O3	0.085 (2)	0.0503 (15)	0.0244 (12)	0.0198 (15)	−0.0099 (12)	−0.0062 (11)
N2	0.0453 (16)	0.0324 (15)	0.0220 (12)	−0.0026 (13)	−0.0077 (11)	0.0010 (11)
C8	0.0286 (17)	0.0346 (18)	0.0233 (14)	−0.0032 (14)	0.0019 (12)	0.0037 (12)
C9	0.0314 (17)	0.0344 (18)	0.0240 (15)	−0.0012 (14)	−0.0021 (12)	0.0000 (13)
C10	0.0269 (17)	0.0427 (18)	0.0182 (12)	−0.0047 (14)	−0.0033 (12)	0.0011 (13)
C11	0.0304 (17)	0.0335 (19)	0.0312 (16)	0.0005 (15)	−0.0003 (13)	0.0033 (14)
C12	0.0384 (19)	0.0336 (18)	0.0337 (16)	0.0003 (15)	0.0013 (14)	−0.0027 (14)
C13	0.0346 (18)	0.0376 (18)	0.0232 (14)	−0.0045 (15)	−0.0032 (13)	−0.0017 (13)
N3	0.0336 (15)	0.0445 (18)	0.0251 (12)	−0.0069 (14)	−0.0066 (11)	0.0053 (13)
O4	0.0796 (19)	0.0431 (15)	0.0297 (12)	−0.0025 (14)	−0.0110 (12)	−0.0067 (11)
O5	0.0541 (16)	0.0731 (19)	0.0356 (12)	0.0159 (15)	−0.0163 (11)	0.0044 (13)

### Geometric parameters (Å, °)

**Table:**

C1—C2	1.405 (5)	N2—C8	1.426 (4)
C1—C6	1.415 (4)	N2—H2N	0.8800
C1—C7	1.520 (4)	C8—C13	1.407 (4)
C2—C3	1.403 (4)	C8—C9	1.407 (4)
C2—H2	0.9500	C9—C10	1.391 (4)
C3—C4	1.399 (4)	C9—H9	0.9500
C3—H3	0.9500	C10—C11	1.391 (4)
C4—C5	1.393 (4)	C10—N3	1.493 (4)
C4—N1	1.494 (4)	C11—C12	1.401 (4)
C5—C6	1.398 (4)	C11—H11	0.9500
C5—H5	0.9500	C12—C13	1.390 (4)
C6—H6	0.9500	C12—H12	0.9500
N1—O2	1.232 (4)	C13—H13	0.9500
N1—O1	1.240 (4)	N3—O5	1.230 (3)
C7—O3	1.235 (4)	N3—O4	1.240 (4)
C7—N2	1.378 (4)		
			
C2—C1—C6	120.0 (3)	C7—N2—C8	127.6 (3)
C2—C1—C7	116.4 (2)	C7—N2—H2N	116.2
C6—C1—C7	123.5 (3)	C8—N2—H2N	116.2
C3—C2—C1	120.3 (3)	C13—C8—C9	119.5 (3)
C3—C2—H2	119.8	C13—C8—N2	117.9 (2)
C1—C2—H2	119.8	C9—C8—N2	122.6 (3)
C4—C3—C2	118.1 (3)	C10—C9—C8	117.4 (3)
C4—C3—H3	120.9	C10—C9—H9	121.3
C2—C3—H3	120.9	C8—C9—H9	121.3
C5—C4—C3	123.0 (3)	C9—C10—C11	124.7 (2)
C5—C4—N1	118.9 (2)	C9—C10—N3	117.7 (3)
C3—C4—N1	118.1 (3)	C11—C10—N3	117.6 (3)
C4—C5—C6	118.4 (3)	C10—C11—C12	116.7 (3)
C4—C5—H5	120.8	C10—C11—H11	121.6
C6—C5—H5	120.8	C12—C11—H11	121.6
C5—C6—C1	120.2 (3)	C13—C12—C11	120.9 (3)
C5—C6—H6	119.9	C13—C12—H12	119.6
C1—C6—H6	119.9	C11—C12—H12	119.6
O2—N1—O1	123.7 (3)	C12—C13—C8	120.9 (3)
O2—N1—C4	118.6 (3)	C12—C13—H13	119.6
O1—N1—C4	117.7 (3)	C8—C13—H13	119.6
O3—C7—N2	123.0 (3)	O5—N3—O4	123.1 (3)
O3—C7—C1	120.2 (3)	O5—N3—C10	118.4 (3)
N2—C7—C1	116.8 (3)	O4—N3—C10	118.5 (3)
			
C6—C1—C2—C3	1.4 (4)	O3—C7—N2—C8	−3.5 (5)
C7—C1—C2—C3	177.5 (3)	C1—C7—N2—C8	175.1 (3)
C1—C2—C3—C4	−1.2 (5)	C7—N2—C8—C13	178.0 (3)
C2—C3—C4—C5	0.6 (5)	C7—N2—C8—C9	−3.1 (5)
C2—C3—C4—N1	179.8 (3)	C13—C8—C9—C10	0.1 (4)
C3—C4—C5—C6	−0.1 (5)	N2—C8—C9—C10	−178.7 (3)
N1—C4—C5—C6	−179.3 (3)	C8—C9—C10—C11	0.4 (4)
C4—C5—C6—C1	0.2 (5)	C8—C9—C10—N3	178.9 (3)
C2—C1—C6—C5	−0.8 (5)	C9—C10—C11—C12	−0.5 (4)
C7—C1—C6—C5	−176.7 (3)	N3—C10—C11—C12	−179.0 (3)
C5—C4—N1—O2	169.1 (3)	C10—C11—C12—C13	0.2 (5)
C3—C4—N1—O2	−10.1 (4)	C11—C12—C13—C8	0.3 (5)
C5—C4—N1—O1	−11.0 (4)	C9—C8—C13—C12	−0.4 (5)
C3—C4—N1—O1	169.8 (3)	N2—C8—C13—C12	178.4 (3)
C2—C1—C7—O3	−19.7 (5)	C9—C10—N3—O5	−165.8 (3)
C6—C1—C7—O3	156.3 (3)	C11—C10—N3—O5	12.8 (4)
C2—C1—C7—N2	161.6 (3)	C9—C10—N3—O4	13.9 (4)
C6—C1—C7—N2	−22.4 (4)	C11—C10—N3—O4	−167.5 (3)

### Hydrogen-bond geometry (Å, °)

**Table:**

*D*—H···*A*	*D*—H	H···*A*	*D*···*A*	*D*—H···*A*
C2—H2···O1^i^	0.95	2.50	3.375 (4)	153.
C5—H5···O3^ii^	0.95	2.38	3.263 (4)	155.
C13—H13···O5^iii^	0.95	2.51	3.421 (4)	162.

Symmetry codes: (i) *x*, *y*, *z*+1; (ii) *x*, *y*, *z*−1; (iii) *x*−1, *y*, *z*−1.

## References

[bb1] Adams, H., Bernad, P. L. Jr, Eggleston, D. S., Haltiwanger, R. C., Harris, K. D. M., Hembury, G. A., Hunter, C. A., Livingstone, D. J., Kariuki, B. M. & McCabe, J. F. (2001). *Chem. Commun.* pp. 1500–1501.

[bb2] Bruker (2009). *APEX2*, *GIS*, *SAINT*, and *SADABS* Bruker AXS Inc., Madison, Wisconsin, USA.

[bb3] Cason, C. J. (2004). *POV-RAY* Persistence of Vision Raytracer Pty Ltd, Williamstown, Australia.

[bb4] Dolomanov, O. V., Bourhis, L. J., Gildea, R. J., Howard, J. A. K. & Puschmann, H. (2009). *J. Appl. Cryst.* **42**, 339–341.

[bb5] Hariharan, M. & Srinivasan, R. (1990). *Acta Cryst.* C**46**, 1056–1058.

[bb6] Kang, S.-B., Yim, H.-S., Won, J.-E., Kim, M.-J., Kim, J.-J., Kim, H.-K., Lee, S.-G. & Yoon, Y.-J. (2008). *Bull. Korean Chem. Soc.* **29**, 1025–1032.

[bb7] Kichitaro, T. (1954). *Yakugaku Zasshi*, **73**, 810–817.

[bb8] Macrae, C. F., Bruno, I. J., Chisholm, J. A., Edgington, P. R., McCabe, P., Pidcock, E., Rodriguez-Monge, L., Taylor, R., van de Streek, J. & Wood, P. A. (2008). *J. Appl. Cryst.* **41**, 466–470.

[bb9] Novozhilova, N. V., Magomedova, N. S., Tudorovskaya, G. L. & Bel’skii, V. K. (1986). *Zh. Strukt. Khim.* **27**, 169–175.

[bb10] Pidcock, E. (2005). *Chem. Commun.* pp. 3457–3459. 10.1039/b505236j 15997296

[bb11] Shchel’tsyn, V. K., Vaisman, I. L., Gitis, S. S., Kozhevnikova, N. L. & Ovchinnikova, L. A. (1972). *Sin. Anal. Strukt. Org. Soedin.* **4**, 46–50.

[bb12] Sheldrick, G. M. (2008). *Acta Cryst.* A**64**, 112–122. 10.1107/S0108767307043930 18156677

[bb13] Spek, A. L. (2009). *Acta Cryst.* D**65**, 148–155. 10.1107/S090744490804362X PMC2631630 19171970

[bb14] Sun, Y., Wang, G. & Guo, W. (2009). *Tetrahedron*, **65**, 3480–3485.

[bb15] Westrip, S. P. (2010). *J. Appl. Cryst.* **43**, 920–925.

